# Role of *ACLY* in the development of gastric cancer under hyperglycemic conditions

**DOI:** 10.1002/qub2.36

**Published:** 2024-03-01

**Authors:** Keran Sun, Jingyuan Ning, Keqi Jia, Xiaoqing Fan, Hongru Li, Jize Ma, Meiqi Meng, Cuiqing Ma, Lin Wei

**Affiliations:** ^1^ Department of Immunology Immunology Department of Hebei Medical University Shijiazhuang China; ^2^ Department of Pathology Pathology Department of the Fourth Hospital of Hebei Medical University Shijiazhuang China

**Keywords:** *ACLY*, gastric cancer, high glucose, immune microenvironment

## Abstract

To investigate the impact of hyperglycemia on the prognosis of patients with gastric cancer and identify key molecules associated with high glucose levels in gastric cancer development, RNA sequencing data and clinical features of gastric cancer patients were obtained from The Cancer Genome Atlas (TCGA) database. High glucose‐related genes strongly associated with gastric cancer were identified using weighted gene co‐expression network and differential analyses. A gastric cancer prognosis signature was constructed based on these genes and patients were categorized into high‐ and low‐risk groups. The immune statuses of the two patient groups were compared. ATP citrate lyase (*ACLY*), a gene significantly related to the prognosis, was found to be upregulated upon high‐glucose stimulation. Immunohistochemistry and molecular analyses confirmed high ACLY expression in gastric cancer tissues and cells. Gene Set Enrichment Analysis (GSEA) revealed the involvement of *ACLY* in cell cycle and DNA replication processes. Inhibition of *ACLY* affected the proliferation and migration of gastric cancer cells induced by high glucose levels. These findings suggest that *ACLY*, as a high glucose‐related gene, plays a critical role in gastric cancer progression.

## 1 INTRODUCTION

Gastric cancer is a prevalent malignancy, ranking as the fifth most common type of neoplasm worldwide and the fourth leading cause of cancer‐related mortality [1]. *Helicobacter pylori* and Epstein–Barr virus infection, smoking, and genetic factors are primary risk factors for gastric cancer [2]. The increased occurrence of gastric cancer in certain regions may be linked to dietary patterns [3]. Hyperglycemia is also considered a potential factor contributing to the occurrence of gastric cancer. In the meta‐analyses conducted by Tian et al. [4] and Yoon et al. [5], a high risk of gastric cancer was found in patients with diabetes. In 2013, Chocarro‐Calvo et al. [6] reported that hyperglycemia could increase β‐catenin acetylation, leading to enhanced Wnt signaling in cancer cells. This finding may explain the high incidence of obesity and diabetes‐related cancers. In 2020, Yang et al. [7] reported results from a retrospective cohort study of 195,312 adults who had undergone upper gastrointestinal endoscopy over 10 years, demonstrating an elevated risk of gastric cancer in patients with diabetes. These studies collectively suggest that hyperglycemia is critically involved in gastric cancer development.

In addition to increasing the incidence of gastric cancer, hyperglycemia accelerates the risk of mortality in patients with gastric cancer [8]. Although not all studies support this association, the majority of observational studies have suggested an increased risk of gastric cancer among individuals with diabetes, particularly among women and Asians. Additionally, patients with gastric cancer and diabetes experience more complications and a poor prognosis following gastrectomy or chemotherapy [9]. In 2011, Tseng et al. [10] conducted an analysis of relevant risk factors for mortality due to gastric cancer in the general population of the Taiwan region of China between 1995 and 2006. Their results revealed that patients with gastric cancer and diabetes had a high mortality rate. They concluded that type 2 diabetes‐induced hyperglycemia and insulin resistance are the underlying causes of the high mortality rate observed in patients with gastric cancer [8].

Currently, the mechanism by which diabetes may act as a potential risk factor for gastric cancer is not fully understood. However, some studies suggest that glucose itself may affect cancer development through increased β‐catenin acetylation and enhanced Wnt signaling pathway [6]. Additionally, the expression of pro‐inflammatory cytokines (such as interleukin‐1, interleukin‐6, and tumor necrosis factor‐α) may be increased in diabetic patients, which may upregulate and activate the Wnt/β‐catenin pathway [11, 12]. Animal studies support the evidence that hyperglycemia and/or hyperinsulinemia enhance N‐methyl‐N‐nitrosourea‐induced stomach carcinogenesis in diabetic (db/db) mice [13]. Furthermore, hyperglycemia may promote carcinogenesis by increasing the expression of reactive oxygen species that cause DNA damage [14], or by increasing the expression of vascular endothelial growth factors associated with tumor angiogenesis and metastasis [15]. Additionally, hyperglycemia may impair immune function, leading to susceptibility to *H. pylori* infection and delayed healing of gastric ulcers after *H. pylori* infection [8]. Finally, high sugar levels may promote the Warburg effect in tumor cells, which favors cancer initiation and progression [8].

The association between diabetes and gastric cancer has been established; however, the underlying mechanism remains elusive. Thus, we aimed to identify key molecules that are associated with high glucose levels and contribute to gastric cancer development. Moreover, we intended to provide insights into the functional mechanisms underlying this association.

## 2 RESULTS

### 2.1 Screening and functional enrichment analysis of high glucose‐related differentially expressed genes

**FIGURE 1:**
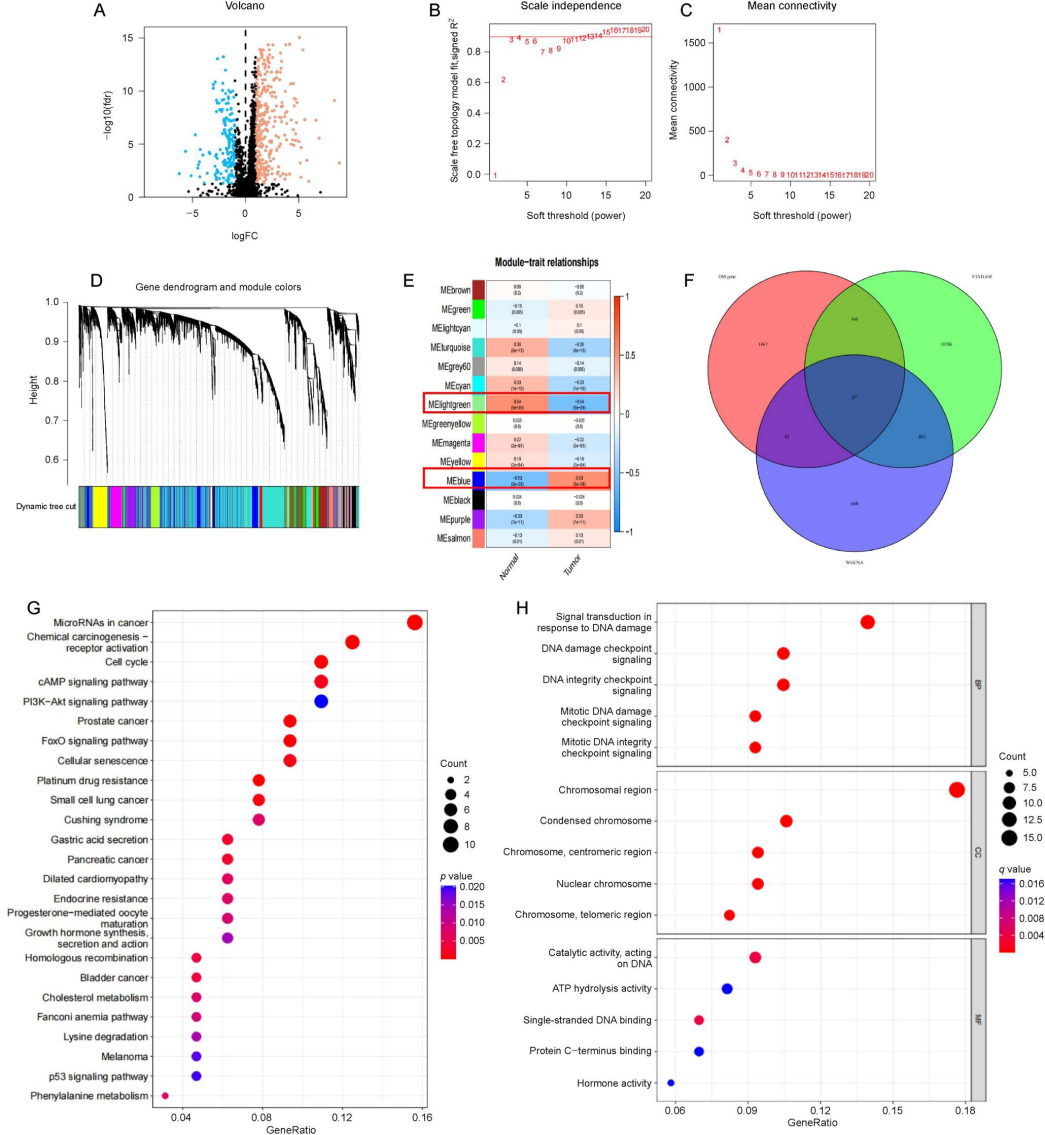
(A) Differential expression of high glucose‐related genes in gastric cancer. (B, C) Determining the optimal soft threshold. (D) Gene clustering. (E) Correlation and significance between computational modules and tumors. (F) Co‐expression of high glucose‐related differential genes in gastric cancer. (G) The Kyoto Encyclopedia of Genes and Genomes enrichment. (H) Gene Ontology enrichment.

We initially collected genes previously reported to be modulated by high‐glucose stimulation and referred to them as high glucose‐related genes. On performing differential expression analysis of these genes in gastric cancer tissue compared with those in normal tissue using the TCGA database, we identified 482 high glucose‐related differentially expressed genes (DEGs) in gastric cancer tissue (Figure 1A). Subsequently, we conducted a weighted gene co‐expression network analysis (WGCNA) and selected the module with the highest co‐expression coefficient for further analysis (Figure 1B–E). Through the intersection of the genes obtained from the WGCNA, we identified 87 co‐expressed high glucose‐related DEGs (Figure 1F). To gain further insights into these 87 DEGs, we performed an enrichment analysis on them. The Kyoto encyclopedia of genes and genomes (KEGG) enrichment analysis revealed that the DEGs were primarily enriched in microRNAs in cancer, chemical carcinogenesis‐receptor activation, cell cycle, and cAMP signaling pathways (Figure 1G). The Gene Ontology (GO) enrichment analysis showed that the DEGs were primarily enriched in pathways involved in signal transduction in response to DNA damage, DNA damage checkpoint signaling, DNA integrity checkpoint signaling, and mitotic DNA damage checkpoint signaling (Figure 1H).

### 2.2 Construction of a predictive signature

**FIGURE 2:**
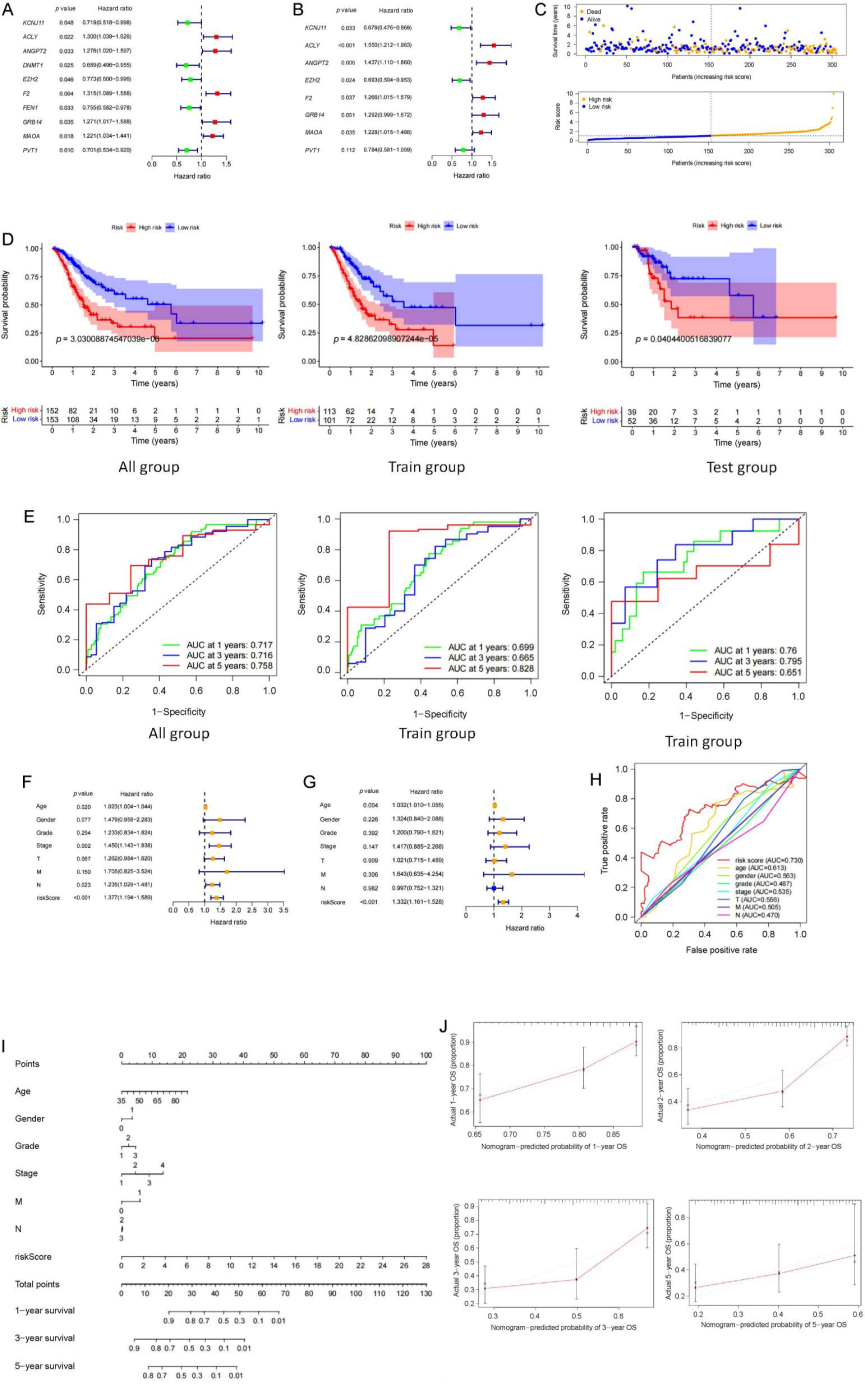
(A) Univariate analysis of prognosis‐related genes. (B) Multivariate analysis of prognostic related genes. (C) Grouped patients according to risk scores. (D) Prognosis of high‐risk versus low‐risk groups. (E) ROC curve for risk score. (F) Univariate analysis of clinical factors. (G) Multivariate analysis of clinical factors. (H) ROC curve for clinical factors. (I) Nomogram. (J) Verifying the accuracy of the nomogram. Immune cell infiltration analysis. (D) Immune function analysis. (E) Immune checkpoint expression analysis.

We performed a univariate analysis using the 87 identified DEGs and the survival time of the patients. The results showed that 10 DEGs were significantly associated with patient prognosis and survival (Figure 2A, *p* < 0.05). Subsequently, we established a prognostic prediction signature for patients with gastric cancer, using the principle of Akaike information criterion (AIC) optimality and incorporating eight genes in the risk calculation score based on multivariate analysis (Figure 2B, *p* < 0.05). Among these genes, *ACLY* exhibited the greatest impact on patient prognosis (*p* < 0.001). The risk scores were then computed for all patients using the expression levels of the eight high glucose‐related DEGs, resulting in the development of the following predictive signature (Table 1).

**TABLE 1:** Multivariate analysis of prognostic related genes.

Gene	*Coef*	HR	HR.95L	HR.95H	*p*‐value
*KCNJ11*	−0.386548791	0.679397576	0.476374747	0.968945289	0.032831677
*ACLY*	0.438365304	1.550171088	1.212118951	1.982503779	0.000478218
*ANGPT2*	0.362706456	1.437213912	1.110399173	1.860217369	0.005859645
*EZH2*	−0.366526189	0.693137984	0.504199663	0.952877006	0.023993813
*F2*	0.235664976	1.265750182	1.014859495	1.578665353	0.03654348
*GRB14*	0.256569447	1.292488522	0.999164348	1.671923724	0.050748728
*MAOA*	0.205260753	1.227845188	1.014869124	1.485515492	0.03470204
*PVT1*	−0.243251623	0.784074198	0.580737827	1.058605655	0.112256153

*Note*: *Coef* represents the coefficient value.

Risk score = (−0.387 × *KCNJ11* expression) + (0.438 × *ACLY* expression) + (0.363 × *ANGPT2* expression) + (−0.367 × *EZH2* expression) + (0.236 × *F2* expression) + (0.257 × *GRB14* expression) + (0.205 × *MAOA* expression) + (−0.243 × *PVT1* expression).

To determine the validity and accuracy of this predictive signature, we randomly divided all patients into training and test groups. The median risk score was used as the threshold value to classify patients into high‐ and low‐risk groups. The risk distribution graph demonstrated an increasing number of patient deaths with high‐risk scores (Figure 2C). Survival analysis for all subgroups indicated that patients in the low‐risk group had significantly better overall survival (OS) than those in the high‐risk group (Figure 2D). The area under the curve (AUC) values in the receiver operating characteristic (ROC) curves were 0.717, 0.716, and 0.758 at 1, 3, and 5 years, respectively. The AUC values were 0.699, 0.665, and 0.828 in the training group and 0.76, 0.795, and 0.651 in the test group at 1, 3, and 5 years, respectively (Figure 2E). These findings indicate that the prognostic signature is accurate and clinically relevant. Furthermore, we examined the association between the risk score, clinical characteristics, and patient survival time. Univariate and multivariate analysis demonstrated that the risk score highly affected patient survival (Figure 2F,G). The ROC curve displayed the largest AUC value for the risk score, reaching 0.730 (Figure 2H). Nevertheless, age, gender, disease grade, and stage can also influence patient prognosis. Therefore, we constructed a nomogram to predict prognosis in patients with gastric cancer by considering clinical features and the risk score (Figure 2I). Calibration curves showed good consistency between the predicted 1‐, 2‐, 3‐, and 5‐year survival rates and actual OS rates of patients (Figure 2J).

### 2.3 Immunosuppressive microenvironment in gastric cancer with high expression of high glucose‐related genes

**FIGURE 3:**
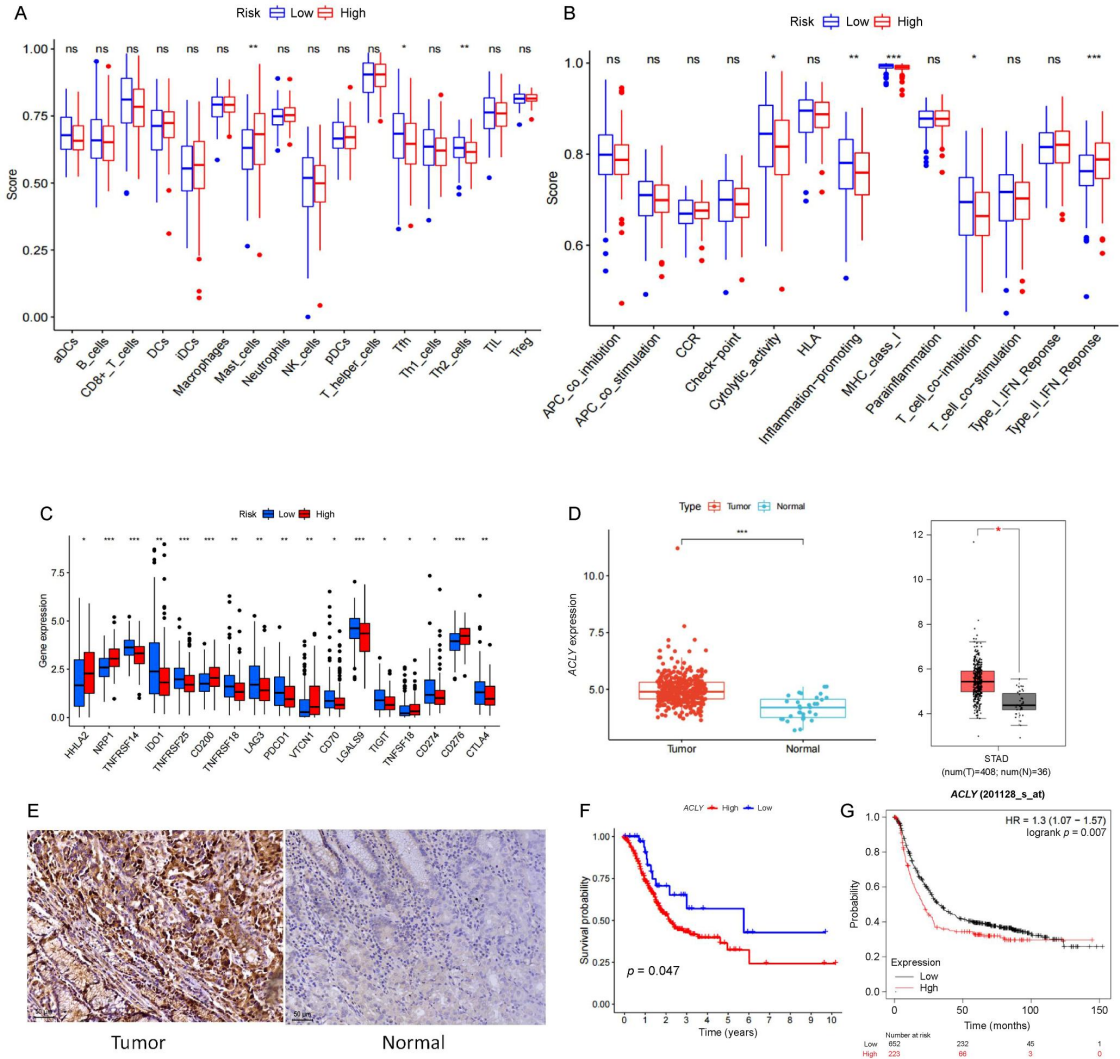
(A) Immune cell infiltration analysis. (B) Immune function analysis. (C) Immune checkpoint expression analysis. (D) Expression of *ACLY* in gastric cancer and adjacent tissues. (TCGA database; GEPIA database). (E) Immunohistochemical staining of ACLY in gastric cancer and adjacent tissues. (F) Effect of *ACLY* on the prognosis of patients with gastric cancer (TCGA database). (G) Effect of *ACLY* on the prognosis of patients with gastric cancer (KM database).

We used the risk score to categorize patients into high‐ and low‐risk groups and observed considerable differences in the immune status of tumor tissue between these two groups. Notably, immune cell infiltration analysis revealed significant differences in the infiltration of the mast and Th2 cells between the high‐ and low‐risk groups (Figure 3A). Immune function analysis further demonstrated significant differences in the expression of MHC‐I molecules and production of type II interferon, indicating a potential inhibition of antigen presentation in the high‐risk group (Figure 3B). However, analysis of immune checkpoints revealed that most immune checkpoints were highly expressed in the high‐risk group, suggesting a suppressed immune microenvironment (Figure 3C). These findings offer valuable insights into the underlying mechanisms that contribute to the prognostic value of our risk score. Additionally, they suggest potential targets for enhancing the immune status of high‐risk group patients.

### 2.4 Effect of *ACLY* on the prognosis of patients with gastric cancer

Using multivariate analysis, we identified *ACLY* as the most critical factor affecting the prognosis of patients with gastric cancer (Figure 2B, *p* < 0.001). Consequently, our research focused on *ACLY*. Initially, we validated the differential expression of *ACLY* in gastric cancer tissues using the TCGA and Gene Expression Profiling Interactive Analysis online databases (Figure 3D). We validated the high expression of *ACLY* in gastric cancer tissue, using immunohistochemistry (Figure 3E). Subsequent survival analysis based on the TCGA database demonstrated that *ACLY* significantly influenced the prognosis and survival rate of patients with gastric cancer (Figure 3F, *p* < 0.05). Moreover, the Kaplan–Meier plotter database analysis demonstrated that *ACLY* was a significant risk‐related factor affecting the prognosis of patients with gastric cancer (Figure 3G, *p* < 0.05).

### 2.5 Correlation of *ACLY* expression with clinical characteristics of patients with gastric cancer

**FIGURE 4:**
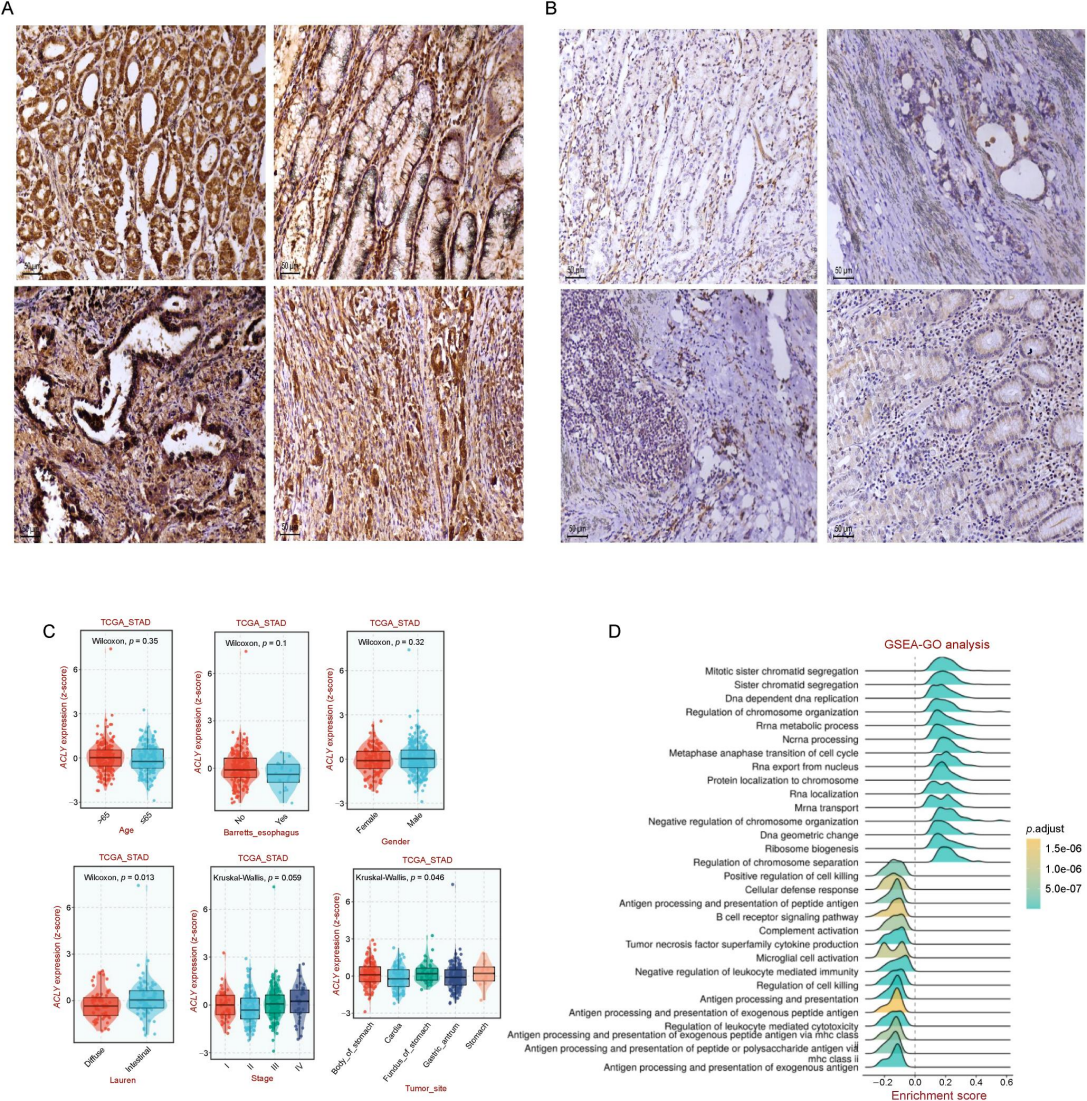
(A) Immunohistochemistry of patients with high expression of ACLY. (B) Immunohistochemistry of patients with low expression of ACLY. (C) Correlation of *ACLY* with clinical features of patients. (D) GSEA GO Analysis.

To investigate the association between *ACLY* expression and clinical characteristics of patients with gastric cancer, we analyzed the correlation between *ACLY* expression and clinical data using both the collected clinical data and the TCGA database. Our clinical data analysis revealed that patients with gastric cancer and diabetes had lower tumor differentiation and higher ACLY expression compared with those without diabetes (Figure 4A,B; Tables 2 and 3). The TCGA analysis showed that *ACLY* expression was not significantly correlated with age, gender, Barrett’s esophagus, or tumor stage. However, *ACLY* expression was associated with Lauren classification and tumor site (Figure 4C). Notably, *ACLY* expression was higher in intestinal‐type gastric cancer than in diffuse‐type gastric cancer in the Lauren classification (Figure 4C, *p* < 0.05). Furthermore, tumors located in the body of the stomach exhibited higher *ACLY* expression than those located in other sites (Figure 4C, *p* < 0.05). In order to explore the relationship between ACLY expression and clinical characteristics of patients, we divided clinical data into patients with high expression of ACLY and patients with low expression of ACLY. Univariate analysis showed no statistically significant differences between ACLY expression levels and patient gender, staging, and grading. ACLY expression level was only associated with diabetes in patients (Table S1, *p* < 0.05).

**TABLE 2:** Univariate analysis of diabetes with clinical features of patients.

		Diabetes		Diabetes ratio	χ^2^	*p*
		Without	With			
Gender	Male	15	17	53.13%	0.625	0.429
	Female	5	3	37.50%		
Family history of malignant tumor	Without	18	15	45.45%	1.558	0.212
	With	2	5	71.43%		
History of smoking	Without	12	14	53.85%	0.440	0.507
	With	8	6	42.86%		
History of drinking	Without	14	12	46.15%	0.440	0.507
	With	6	8	57.14%		
ACLY	Low	12	2	14.29%	10.989	0.001*
	High	8	18	69.23%		
Nervous invasion	Without	11	9	45.00%	0.648	0.421
	With	8	11	57.89%		
Vascular tumor thrombus	Without	7	3	30.00%	2.133	0.144
	With	13	17	56.67%		
Differentiation	High	13	5	27.78%	6.465	0.011*
	Poorly	7	15	68.18%		
T	T1	1	1	50.00%	0.243	0.97
	T2	3	2	40.00%		
	T3	11	12	52.17%		
	T4	5	5	50.00%		
N	N0	4	5	55.56%	0.404	0.939
	N1	6	5	45.45%		
	N2	5	6	54.55%		
	N3	5	4	44.44%		
M	0	20	19	48.72%	1.026	0.311
	1	0	1	100.00%		
Age	＜60	12	14	53.85%	0.440	0.507
	≥60	8	6	42.86%		

*p＜0.05.

**TABLE 3:** Multivariate analysis of diabetes with clinical features of patients.

		B	S.E.	Wals	df	*p*	OR	OR 95% C.I.	
								Floors	Caps
ACLY	High	−2.692	0.952	8.003	1	0.005*	0.068	0.01	0.437
	Low	0					1		
Differentiation	High	1.654	0.642	6.636	1	0.01*	5.228	0.032	0.817
	Poorly	0					1		

### 2.6 Multifunctional effect of *ACLY* on gastric cancer prognosis

**FIGURE 5:**
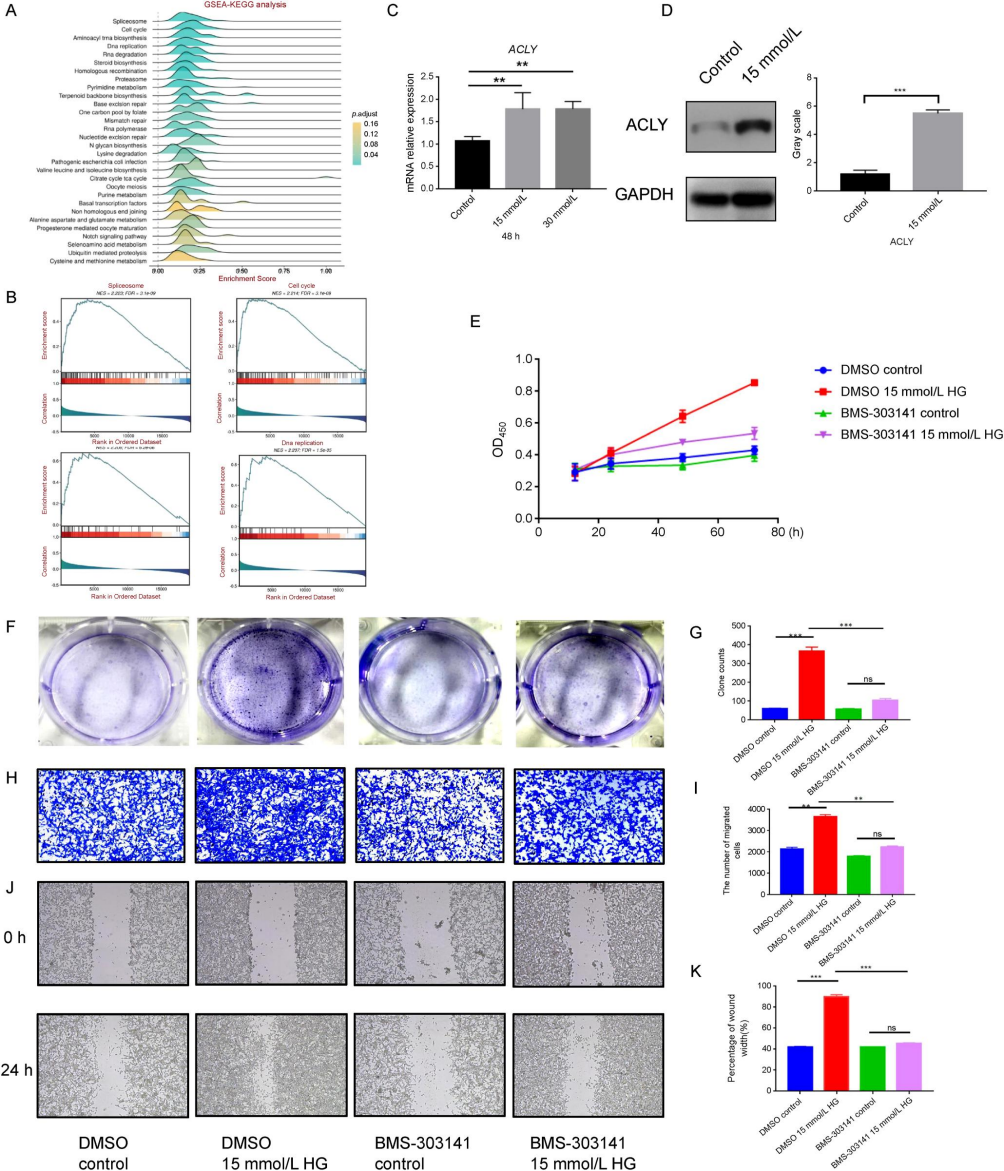
(A) GSEA KEGG Analysis. (B) GSEA KEGG Analysis (specific pathway). (C) qPCR detection of *ACLY* expression in gastric cancer cells stimulated with 15 mmol/L and 30 mmol/L high glucose for 48 h (D) Western blot detection of ACLY expression in gastric cancer cells stimulated with 15 mmol/L high glucose for 48 h. (E) CCK8 experiment was used to detect the cell proliferation activity of MFC cells stimulated with 15 mmol/L high glucose, with or without BMS‐303141 for 24 h, 48 h, and 72 h. (F) The effect of BMS‐303141 on the cloning of gastric cancer cells cultured for 2 weeks in 15 mmol/L high glucose. (G) Data statistics of cloning. (H) Cell migration experiment results of MFC cells in Transwell upper chamber stimulated with 15 mmol/L high glucose (with or without BMS‐303141) for 24 h. (I) Data statistics of cell migration. (J) The effect of BMS‐303141 on wound healing of gastric cancer cells cultured for 2 weeks in 15 mmol/L high glucose. (K) Statistics of wound healing.

To elucidate the mechanisms by which *ACLY* influences the prognosis of patients with gastric cancer, we performed a single‐gene set enrichment analysis using the BEST online analysis tool. The GO enrichment analysis indicated that *ACLY* primarily affects pathways, such as mitotic sister chromatid segregation, sister chromatid segregation, and DNA‐dependent DNA replication (Figure 4D). The KEGG enrichment analysis showed that *ACLY* primarily affects pathways such as spliceosome, cell cycle, aminoacyl tRNA biosynthesis, and DNA replication (Figure 5A). These results indicate that *ACLY* may regulate key biological processes, such as cell cycle and DNA replication, thereby affecting the proliferation activity of gastric cancer cells and ultimately influencing the prognosis of patients with gastric cancer (Figure 5B).

### 2.7 Induction of *ACLY* expression in gastric cancer cells by high glucose

To validate the effect of high glucose on *ACLY* expression in gastric cancer cells, we conducted in vitro experiments by subjecting gastric cancer cells to varying concentrations of glucose and analyzing the RNA expression of *ACLY* via qPCR. Our findings revealed that exposure to high glucose levels of 15 mmol/L significantly increased the RNA expression of *ACLY* (Figure 5C, *p* < 0.05). We further validated the protein expression of ACLY using Western blotting and observed that high glucose levels could elevate its protein expression (Figure 5D).

### 2.8 Inhibition of gastric cancer cell proliferation and metastasis by *ACLY* inhibitor BMS‐303141

To investigate the effect of *ACLY* on the proliferation and metastasis of gastric cancer MFC cells, we employed the ACLY inhibitor BMS‐303141 to inhibit ACLY induced by high glucose levels. The CCK8 experiment demonstrated that BMS‐303141 can suppress the proliferation of gastric cancer cells stimulated by high glucose levels (Figure 5E). Additionally, the cloning experiments exhibited that BMS‐303141 can hinder the cloning of gastric cancer cells induced by high glucose levels (Figure 5F,G; *p* < 0.05). Moreover, the results of the Transwell migration experiment revealed that BMS‐303141 can restrain the migration of gastric cancer cells stimulated by high glucose levels (Figure 5H,I; *p* < 0.05). Furthermore, the wound healing experiment illustrated that BMS‐303141 can impede the migration of gastric cancer cells induced by high glucose levels (Figure 5J,K; *p* < 0.05). Subsequently, we used si‐*ACLY* to knockdown *ACLY* and obtained results similar to those of *ACLY* inhibitor BMS‐303141 (Figure S1). This indicates that the inhibition of *ACLY* can suppress high glucose‐induced proliferation and metastasis of gastric cancer cells.

## 3 DISCUSSION

The incidence of diet‐related diseases, particularly diabetes, is increasing alongside improvements in our standard of living [16]. Diabetes is a risk factor for gastric cancer [8]. Patients with gastric cancer and diabetes have a high risk of death, particularly under conditions of hyperglycemia, which is strongly associated with poor prognosis [9]. Expanding upon these findings, we aimed to elucidate the underlying mechanisms and key molecules implicated in the influence of high glucose levels on gene expression. Our ultimate objective was to uncover potential factors that affect the prognosis of individuals diagnosed with gastric cancer. Our results revealed that high‐glucose stimulation induces overexpression of *ACLY*, a key gene that promotes the occurrence and progression of gastric cancer. The regulatory effect of this molecule may therefore be a primary contributing factor to the poor prognosis observed in patients with gastric cancer and diabetes. In our investigation, we first compiled a list of genes previously reported to be affected by high‐glucose stimulation and subjected them to weighted correlation network and differential analyses. Our findings indicated that these genes were principally enriched in tumor‐related pathways, such as cell‐cycle regulation. These results support the notion that high glucose‐associated genes are significant contributors to tumorigenesis. Furthermore, we performed correlation analysis with patient prognosis information. This identified *ACLY* as a pivotal molecule, linking exposure to high glucose levels to the prognosis of patients with gastric cancer.

In our study, using both single‐factor and multi‐factor COX regression analysis, we identified *KCNJ11, ACLY, ANGPT2, EZH2, F2, GRB14, MAOA,* and *PVT1* as the most relevant genes associated with the prognosis of patients with gastric cancer. Reportedly, these genes are significantly correlated with the development and progression of gastric cancer. *ACLY* encodes acetyl‐coenzyme A synthetase, which plays a role in cell metabolism, and its overexpression has been linked to gastric cancer occurrence and progression [17]. *ANGPT2* encodes a vascular growth involved in angiogenesis regulation, and its overexpression has been associated with gastric cancer angiogenesis, invasion, and metastasis [18, 19]. The overexpression of *EZH2*, which encodes a multifunctional protein involved in gene transcription regulation, has been linked to tumor occurrence and metastasis in gastric cancer [20, 21]. Furthermore, *F2* encodes prothrombin, and its polymorphisms have been associated with gastric cancer development [22]. The low expression level of *GRB14*, which encodes a signaling protein, has been linked to gastric cancer occurrence and progression [23]. *MAOA* encodes an enzyme involved in neurotransmitter metabolism, and its polymorphisms have been associated with gastric cancer development [24]. Moreover, *PVT1* encodes a long non‐coding RNA involved in tumor occurrence and progression, and its overexpression has been associated with gastric cancer development and progression [25, 26, 27]. These genes may all contribute to the development and progression of gastric cancer, but specific research is required to determine how relevant they are:

We divided patients with gastric cancer into high‐ and low‐risk groups based on diabetes‐associated genes and observed significant differences in the infiltration of mast and Th2 cells in the tumor microenvironment. These cells have emerged as key regulators of the immune microenvironment in gastric cancer. Recent studies have uncovered their involvement in various mechanisms that influence tumor progression. Mast cells, which are abundant in the tumor microenvironment, promote angiogenesis, growth, and invasion in gastric cancer by secreting pro‐angiogenic and pro‐inflammatory mediators, such as VEGF, TNF‐α, and IL‐6 [28]. Moreover, mast cells recruit other immune cells such as T cells and macrophages, further enhancing the pro‐inflammatory environment [29]. Similarly, Th2 cells facilitate tumor growth and metastasis by secreting cytokines, such as IL‐4, IL‐5, and IL‐13, which stimulate tumor cell proliferation, angiogenesis, and invasion while inhibiting the function of cytotoxic T and natural killer cells [30]. Notably, the immune status of patients with high‐ and low‐risk gastric cancer are reported to contrast tumor tissues [31]. Particularly, we observed significant differences in the infiltration of the mast and Th2 cells between the high‐ and low‐risk groups, suggesting that the immune microenvironment in high‐risk patients may be more pro‐inflammatory and less immunosuppressive than that in the low‐risk patients. Further analysis of immune function in high‐risk patients revealed significant differences in MHC‐I molecule expression and type II interferon synthesis, indicating a possible inhibition of antigen presentation that could contribute to the development of highly aggressive tumors [32]. Immune checkpoint analysis demonstrated high expression of the most relevant immune checkpoints in the high‐risk group, suggesting that the immune microenvironment in patients with high‐risk gastric cancer was in a suppressed state [33]. These findings emphasize the complexity of the immune microenvironment in gastric cancer and highlight the interplay between different immune cell types and signaling pathways that regulate tumor progression [34]. In summary, mast and Th2 cells play a crucial role in influencing the tumor microenvironment and regulating gastric cancer progression. Further studies are required to elucidate the specific mechanisms through which these cells contribute to gastric cancer progression and to develop novel immunotherapeutic strategies to target these cells.

In our study, *ACLY* was identified as the gene most strongly associated with patient prognosis after analyzing the genes related to patient prognosis. *ACLY*, as the primary gene involved in de novo fatty acid synthesis, can promote the occurrence and development of gastric cancer through the mevalonate pathway [35]. Moreover, Zheng et al. [36] conducted a bioinformatics analysis of data on patients with gastric cancer from the TCGA database and found *ACLY* to be the most significant differentially expressed gene associated with patient prognosis. The expression of *ACLY* was strongly correlated with patient prognosis, indicating its potential as a crucial target for the treatment of gastric cancer. Furthermore, Cheng et al. [37] performed experiments at the cellular level to analyze *ACLY* expression in human gastric cancer tissues. They observed that inhibiting *ACLY* expression and activation reduced the proliferation and invasion of gastric cancer cells, and *ACLY* was found to inhibit gastric cancer cell proliferation by regulating the expression of peroxisome proliferator‐activated receptor‐γ. These findings are consistent with bioinformatic analysis results, validating *ACLY* as a key therapeutic target for gastric cancer. Additionally, Liu et al. [38] conducted a metabolomic analysis of trastuzumab‐resistant gastric cancer cells and found a significant correlation between *ACLY* expression and trastuzumab resistance in patients with gastric cancer.

These studies collectively demonstrate that hyperglycemia can promote the development of gastric cancer by upregulating *ACLY* expression. *ACLY* not only plays a crucial role in promoting the occurrence and development of gastric cancer but also contributes to trastuzumab resistance. Furthermore, *ACLY* has been implicated in various tumors (Table 4). The study by Cao et al. in 2017 confirmed that there are changes in *ACLY* expression in mice with diabetes induced by high glucose intake, indicating that sugar intake can lead to changes in *ACLY* expression in animal bodies [39]. Bradshaw et al. reported in 2021 that fasting can reduce *ACLY* expression levels [40]. In addition, in the study by Hong et al. in 2022, it was found that changes in glucose levels can also cause changes in *ACLY* expression levels in liver cancer cell lines, indicating that sugar intake or changes in sugar metabolism can also affect *ACLY* changes in tumor cells [41]. *ACLY* is highly expressed in many tumors, and this high expression indicates poor prognosis, increased tumor activity, and increased metastasis in patients. Therefore, controlling sugar intake is particularly important for tumor patients, especially those with diabetes.

**TABLE 4:** The role of *ACLY* in various tumors.

Cancer	The role of *ACLY*	Refs
Gastric cancer	*ACLY* influences gastric cancer cell proliferation and metastasis by regulating fatty acid metabolism;	[35]
	WCGNA analysis shows that *ACLY* is significantly associated with the prognosis of gastric cancer patients;	[36]
	*ACLY* inhibits gastric cancer cell proliferation by regulating the expression of peroxisome proliferator‐activated receptor‐γ;	[37]
	*ACLY* promotes trastuzumab resistance in gastric cancer cells	[38]
Lung cancer	CUL3 inhibits lipid synthesis, cell proliferation and tumor growth in lung cancer cells by targeting *ACLY* via KLHL25;	[45]
	Epithelial‐mesenchymal transition of bronchial epithelial cells induced by high expression of *ACLY* induced by environmental fine particles	[46]
Colon cancer	*ACLY* increases the migratory and invasive ability of colon cancer cells by promoting the translocation of CTNNB1 protein to the nucleus to increase its transcriptional activity;	[47]
	*ACLY* regulates the epithelial‐to‐mesenchymal transition of colon cancer cells by regulating the Wnt‐β‐catenin pathway, thereby causing tumor development;	[48]
	HSPC111 alters lipid metabolism in rectal cancer cells by phosphorylating *ACLY*, excretion of cancer cell exosomes and promotes liver metastasis	[49]
Breast cancer	The expression level of *ACLY* is associated with prognosis, ER status, PR status, tumor size, TNM stage and lymph node invasion in breast cancer patients;	[50]
	The overexpression of *ACLY* is significantly correlated with the expression of multidrug resistance proteins; *ABCB1*/*ABCG2* promotes the drug resistance of breast cancer cells;	[50]
	High expression level of *ACLY* can cause stemness and metastasis of breast cancer	[51]
Ovarian cancer	Knockdown of *ACLY* inhibits cell proliferation by regulating the P16‐CDK4‐CCND1 pathway;	[52]
	Upregulation of *ACLY* correlates with activation of the PI3K‐AKT pathway in cisplatin‐resistant ovarian cancer cell lines;	[52]
	Knockdown of *ACLY* inhibits PI3K‐AKT pathway and induces apoptosis in ovarian cancer cells	[52]
Pancreatic cancer	*ACLY* can promote the occurrence and development of pancreatic cancer through the mevalonate pathway;	[53]
	Both chemical inhibitors of *ACLY* and siRNA approaches targeting *ACLY* inhibit the viability of pancreatic cancer cells	[54]
Prostate cancer	*ACLY* promotes the occurrence and development of prostate cancer by mediating lipid metabolism disorder;	[55, 56]
	*ACLY* can mediate drug resistance in prostate cancer by regulating androgen receptor expression;	[57]
	*ACLY* is a potential target for the treatment of prostate cancer	[58]
Hepatic cancer	*ACLY* promotes the occurrence and development of hepatocellular carcinoma by mediating lipid metabolism disorder;	[59, 60, 61, 62]
	Both *ACLY* chemical inhibitors and *ACLY*‐targeting siRNA approaches inhibit the proliferation and migration and invasion abilities of hepatoma cells;	[63]
	*ACLY* is downregulated in HepG2 and Huh7 cells, but downregulated in Hep3B and HCC‐LM3 cells;	[64]
	Patients with hepatocellular carcinoma with high *ACLY* expression have a lower overall survival rate. AFP level, TNM stage, tumor size and *ACLY* expression level are independent risk factors affecting their overall survival	[65]
	Combination of *ACLY* inhibitor and programmed death ligand 1 antibody largely inhibits chemically induced hepatocarcinogenesis;	[66]
	*ACLY* can regulate the canonical Wnt pathway by affecting the stability of β‐catenin;	[67]
	ONECUT2‐FGF2‐*ACLY* axis regulates metastasis of hepatocellular carcinoma;	[65]
	Bioinformatics analysis shows that *ACLY* is significantly correlated with immune checkpoint CD276 and immune cell infiltration;	[68]
	Downregulation of *ACLY* can reverse sorafenib resistance, and targeting *ACLY* can effectively treat drug‐resistant hepatocellular carcinoma	[69]
Malignant tumors of the head and neck	*ACLY* plays a key role in oral carcinogenesis;	[70]
	*ACLY* affects DNA damage repair, and *ACLY* expression can predict the efficacy of radiotherapy for head and neck squamous cell carcinoma;	[71]
	Long non‐coding RNA TINCR can bind *ACLY* to protect it from ubiquitination degradation, thereby maintaining the level of acetyl‐CoA, promoting the progression of nasopharyngeal carcinoma and chemotherapy resistance	[72]

In the final part of our study, we conducted an immune cell infiltration analysis, which revealed a correlation between *ACLY* and the extent of infiltration by different immune cells. Notably, we found a negative correlation between *ACLY* expression and infiltration of CD8^+^ T cells. Previous studies have highlighted the crucial role of CD8^+^ T cell infiltration in determining the prognosis of patients with gastric cancer [42, 43], providing further evidence of the impact of *ACLY* on patient outcomes. These findings suggest that patients with gastric cancer and high *ACLY* expression may exhibit a tumor microenvironment resembling that of a “cold” tumor which with immunosuppressive microenvironment [44], indicating potential limitations in the effectiveness of immunotherapy in these individuals.

In this study, we aimed to elucidate the mechanisms and key molecules involved in the effect of hyperglycemia on the prognosis of patients with gastric cancer. Among the various molecules considered, *ACLY* stands out as a pivotal molecule regulated by high glucose levels, exerting a substantial influence on the progression of gastric cancer.

## 4 CONCLUSION

The high glucose‐related gene *ACLY* is a key molecule that affects the progression of gastric cancer. High glucose can induce high expression of *ACLY* in gastric cancer cells and enhance their proliferation and migration. Blocking *ACLY* can inhibit high glucose induced proliferation and migration of gastric cancer cells.

## 5 MATERIALS AND METHODS

### 5.1 Data download

The corresponding clinical and prognostic data were downloaded from The Cancer Genome Atlas (TCGA) gastric cancer dataset. The data used in this study were derived from the Transcriptome RNA‐sequencing (RNA‐seq) dataset available on the website of The Cancer Genome Atlas (TCGA). Specifically, we obtained the Fragments Per Kilobase of transcript per Million (FPKM) mapped reads, which is a normalized measure of gene expression levels. The FPKM values provide an estimate of the abundance of each transcript in the sample, taking into account both the length of the transcript and the total number of reads mapped to it. By utilizing this standardized RNA‐seq data, we were able to accurately assess the gene expression profiles in our analysis, ensuring reliable and representative results. FPKM values were transformed into transcripts per million (TPM). The RNA expression values of 343 gastric cancer tissues and 30 normal tissues were obtained, as well as the survival time and clinical data of gastric cancer patients. A summary of 2032 high glucose‐related genes (Table S2) was compiled from the National Center for Biotechnology Information (NCBI), GeneCards, Online Mendelian Inheritance in Man (OMIM), and KEGG.

### 5.2 Differential expression analysis, enrichment analysis and construction of a predictive signature

High glucose‐related DEGs were identified using screening criteria of |log2 fold change (FC)| > 1 and an adjusted *p*‐value <0.05. Differential expression analysis was performed using the “limma” package, while enrichment analysis was conducted using the “GSEABase,” “ClusterProfiler,” and “org.Hs. for example,.db” packages. The resulting data were visualized using the “ggplot2” and “ggpubr” packages. Univariate Cox analysis, least absolute shrinkage and selection operator (LASSO) regression analysis and multivariate Cox analysis were used to identify entosis‐related lncRNA linked to patients’ prognosis. Risk scores are calculated for all patients based on the following formula:

$$\text{Risk}\;\text{score}=\sum_{i=1}^{n}\left({Coef}_{i}\times x_{i}\right)$$

*Coef* represents the coefficient value, and x represents the selected nine entosis‐related lncRNAs. This formula was used to calculate the risk score for each patient.

### 5.3 Cell culture

The human gastric cancer cell line MGC‐803 was thawed and revived using standard procedures. Cells were cultured in RPMI1640 medium supplemented with 10% heat‐inactivated fetal bovine serum at 37°C in a 5% CO_2_ incubator, with a change of medium every three days. Once the cells reached stable passages, logarithmic growth phase cells were selected for subsequent experiments. Transfection of siRNA was performed using lipofectamine 2000 (Invitrogen, USA). The primer sequences for si‐*ACLY* and si‐NC are provided in Table S3.

### 5.4 Cell stimulation and RT‐PCR

5 × 10^5^ MGC‐803 cells were seeded in six‐well plates until they adhered. The cells were washed three times with phosphate buffer solution before stimulation. The culture medium was replaced with serum‐free RPMI1640 before the experiment. For each experimental group, cells were stimulated with 15 mmol/L and 30 mmol/L high glucose, while an equal volume of RPMI1640 was used as the control group. Six hours later, cells were harvested for RNA extraction using the Trizol method according to the kit instructions (cwbio, CW2623S) in a 25 μL reaction system. The primer sequences for *ACLY* are provided in Table S3.

### 5.5 Immunohistochemistry (IHC)

Tissue samples fixed in 10% neutral formaldehyde and embedded in paraffin were sectioned into 4 μm slices, dewaxed with xylene, and gradually dehydrated with ethanol. Antigen retrieval was performed by boiling the sections in sodium citrate buffer for 15 min, followed by treatment with 3% hydrogen peroxide for 10 min to remove endogenous enzymes. Each tissue section was then blocked with 50 μL of goat serum for 30 min. After discarding the goat serum, 50 μL of rabbit anti‐human *ACLY* (1:400) monoclonal antibody was added and incubated at 4°C overnight. The primary antibody was discarded the next day and the secondary antibody was added and incubated for 30 min. The tissue sections were treated with DAB chromogenic reagent and observed under a microscope. Post‐hematoxylin counterstaining was performed, followed by dehydration with 70%, 80%, 90%, and 95% absolute ethanol for 5 min each and with xylene for 20 min. Finally, the slides were sealed with neutral resin.

### 5.6 Western blot

The cellular total protein was extracted from each group, and its concentration was determined. An SDS‐PAGE gel with a volume fraction of 12% was prepared, and an equal amount of protein was loaded for electrophoresis and subsequently transferred to a PVDF membrane. The membrane was blocked with 5% skimmed milk at room temperature for 2 hours, followed by incubation with GAPDH (1:1000) and *ACLY* (1:1000) overnight at 4°C on a shaking table. After washing, a secondary antibody with a dilution of 1:2000 was added and incubated at room temperature for 2 hours. Finally, the membrane was incubated with an exposure solution and exposure detected, using an image analysis system.

### 5.7 Clinical data collection

In this retrospective study, we analyzed a cohort of 40 patients with gastric cancer who were treated at The Fourth Hospital of Hebei Medical University between January 2018 and May 2022. Each patient was confirmed to have GC by two pathologists and there was no discrepancy in histological diagnosis. The clinical data extracted from each patient included age, sex, *ACLY* expression, tumor differentiation, smoking history, drinking history, and other relevant information.

### 5.8 Statistical analysis

Statistical analysis was performed using R (version 4.1.2) and the “Bioconductor” packages. The Kaplan‐Meier method and the logrank test were used to evaluate OS in high‐ and low‐risk groups. Student’s *t*‐test (unpaired, two‐tailed) was used to compare two independent groups, while one‐way analysis of variance (ANOVA) was used as a parametric method and the Kruskal‐Wallis test was used as a non‐parametric method for three or more groups. Additionally, Strawberry Perl software (64‐bit) was used for certain analyses.

### 5.9 CCK‐8 assay

In the CCK‐8 assay, MFC cells stimulated by high glucose concentration were inoculated into a 96‐well plate at a density of 2 × 10^3^ cells overnight. Subsequently, 20 μL of CCK8 solution (5 mg/mL) was added and incubated for 4 hours. The optical density at 450 nm was measured using a microplate instrument.

### 5.10 Wound healing assay

Cell migration was evaluated using a wound healing assay following established protocols. MFC cells that had been transfected were cultured in a six‐well plate until they reached 90% confluency. Scratches were created on the cell monolayer using a micropipette, and the cells were rinsed three times with sterile PBS to remove non‐adherent cells. Subsequently, the fresh serum‐free culture medium was added to the wells, and the cells were further cultured. The wound status at both 0‐ and 24‐h post‐scratch was visualized using an X71 inverted microscope (Olympus), and the average distance between cells was calculated using ImageJ software. All experiments were performed in triplicate to ensure statistical rigor.

### 5.11 Colony formation assay

MFC cells were cultured under standard conditions in a six‐well plate. After a 2‐week incubation at 37°C, the plate was washed with cold phosphate buffer saline to remove non‐adherent cells. Subsequently, colony cells were fixed using 4% paraformaldehyde for 15 min and stained with 0.1% crystal violet at room temperature. Imaging and quantification of the colony cells were performed using a non‐optical microscope.

### 5.12 Transwell assay

Cell migration was evaluated using Transwell assays, following a 48‐h treatment period. MFC cells from each group were seeded into the upper chamber of Transwell inserts at a density of 4 × 10^4^ cells/well in a medium containing 5% fetal bovine serum. Additionally, 500 μL of the medium was added to the lower chamber of a 24‐well culture plate. After conventional culture for 24 h, cells on the upper surface of the insert were removed using a cotton swab. Subsequently, the cells were fixed in a 4% paraformaldehyde solution for 10 min at room temperature and stained with a 0.5% crystal violet solution for 15 min. Finally, five randomly selected fields of view were examined under a light microscope to quantify the number of cells that had invaded the sub‐layer membrane chamber.

### 5.13 Tumor microenvironment analysis and immune cell infiltration analysis

The ImmuneScore, StromalScore, and ESTIMATEScore were calculated using the “estimate” package. The degree of immune cell infiltration was calculated using the ssGSEA algorithm.

### 5.14 Nomogram

Risk score and clinicopathological variables, such as age or stage were used to create a nomogram that can predict 1‐, 3‐, and 5‐year survival of gastric cancer patients. A calibration curve was utilized to see if the anticipated survival rate matched the actual survival rate.

**Abbreviations**
*ACLY*
: ATP citrate lyase
AIC: Akaike information criterion
ANOVA: One‐way analysis of variance
AUC: Area under the curve
DEGs: Differentially expressed genes
FPKM: Fragments per kilobase of transcript per million
GSEA: Gene Set Enrichment Analysis
GO: Gene Ontology
IHC: Immunohistochemistry
KEGG: Kyoto encyclopedia of genes and genomes
LASSO: Least absolute shrinkage and selection operator
NCBI: National Center for Biotechnology Information
OMIM: Online Mendelian Inheritance in Man
OS: Overall survival
ROC: Receiver operating characteristic
TCGA: The Cancer Genome Atlas
TPM: Transcripts per million
WGCNA: Weighted gene co‐expression network analysis

## AUTHOR CONTRIBUTIONS

**Keran Sun**: Conceptualization; data curation; formal analysis; writing original draft; writing review & editing. **Lin Wei**: Conceptualization; funding acquisition; writing original draft; writing review & editing. **Jingyuan Ning**: Writing review & editing. **Keqi Jia**: Writing review & editing. **Xiaoqing Fan**: Writing review & editing. **Hongru Li**: Writing – review & editing. **Jize Ma**: Writing review & editing. **Meiqi Meng**: Writing original draft; writing review & editing. **Cuiqing Ma**: Funding acquisition; project administration; writing review & editing.

## CONFLICT OF INTEREST STATEMENT

The authors Keran Sun, Jingyuan Ning, Keqi Jia, Xiaoqing Fan, Hongru Li, Jize Ma, Meiqi Meng, Cuiqing Ma and Lin Wei declare no conflicts of interest regarding this study.

## AVAILABILITY OF DATA AND MATERIALS

All data are available in the main excel or the supplementary materials. Transcriptomic and clinical data for all patients were obtained from TCGA databases.

## ETHICS APPROVAL AND CONSENT TO PARTICIPATE

This article does not contain any studies with human or animal materials performed by any of the authors.

## Supporting information

Supplementary Material
