# Supplementary material for: Role of ACLY in the development of gastric cancer under hyperglycemic conditions
Source: Quant Biol. 2024 Mar 1;12(1):100–16. doi: 10.1002/qub2.36 (PMC12806462; doi:10.1002/qub2.36)
Supplement: Supplementary file 1 — Supplementary Material [file QUB2-12-100-s001.docx]

Supplementary material

FigureS1
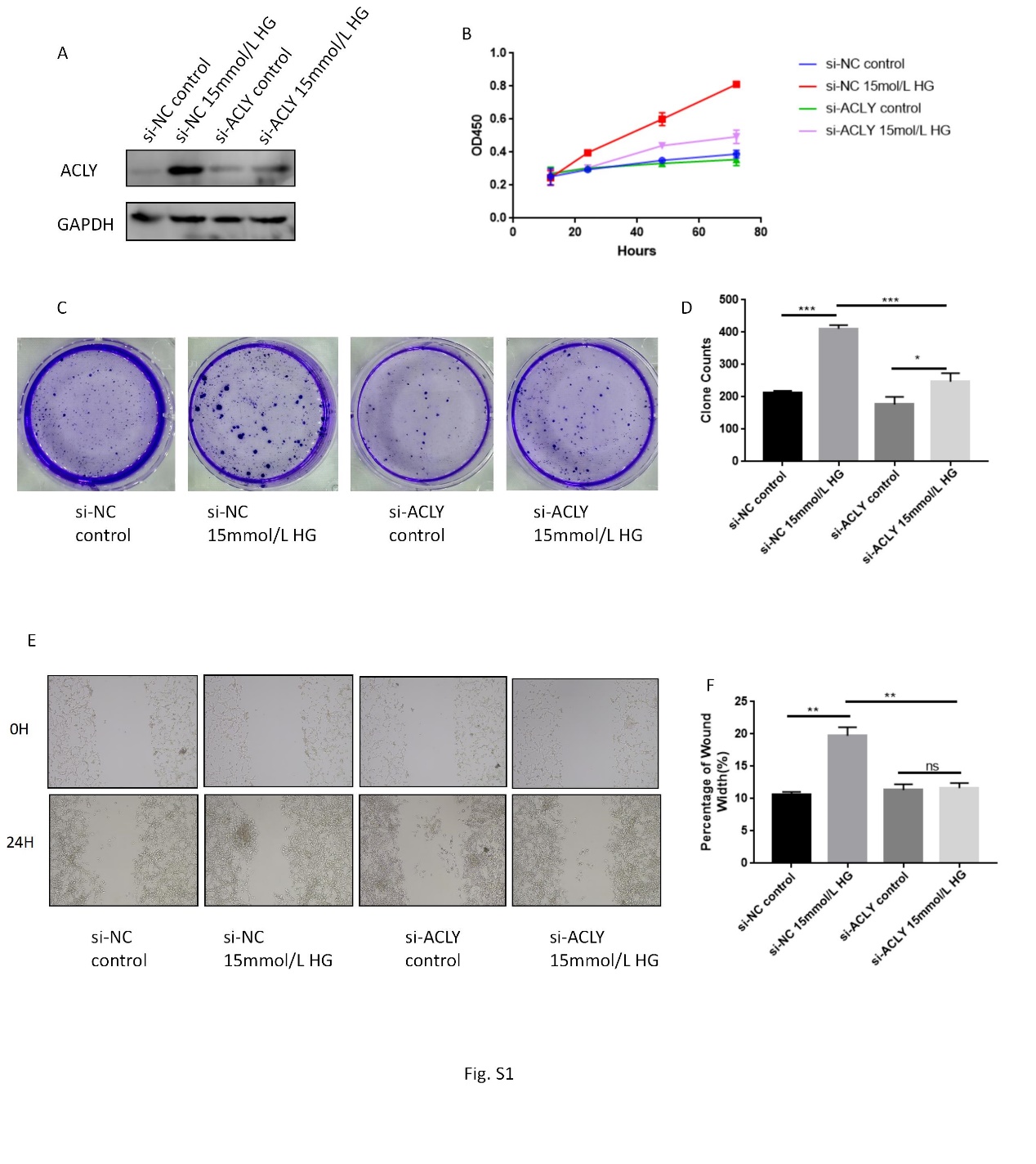


Figure S1 (A) Gastric cancer cells were stimulated with 15 mmol/L high glucose for 48 hours, and the expression of ACLY was detected by Western blot. (B) Use 15 mmol/L high glucose with or without si-ACLY to stimulate MFC cells for 24 hours, 48 hours and 72 hours, and detect OD450 after adding CCK-8 reagent. (C) Gastric cancer cells were cultured for two weeks at 15 mmol/L high glucose with or without si-ACLY, and the cloning of gastric cancer cells was observed by fixed staining. (D) Data statistics of cloning. (E) Gastric cancer cells were cultured at 15 mmol/L high glucose with or without si-ACLY for 24 hours to observe the wound healing of gastric cancer cells. (F) Statistics of wound healing.

**Table S1** Univariate analysis of ACLY with clinical features of patients.

|  |  | ACLY Expression | | Ratio | x^2^ | *P* |
| --- | --- | --- | --- | --- | --- | --- |
|  |  | Low | High |  |  |  |
| Gender | male | 11 | 21 | 65.63% | .027a | 0.868 |
|  | female | 3 | 5 | 62.50% |  |  |
| Family history of malignant tumor | Without | 13 | 20 | 60.61% | 1.600a | 0.206 |
|  | With | 1 | 6 | 85.71% |  |  |
| History of smoking | Without | 10 | 16 | 61.54% | .391a | 0.532 |
|  | With | 4 | 10 | 71.43% |  |  |
| History of drinking | Without | 8 | 18 | 69.23% | .584a | 0.445 |
|  | With | 6 | 8 | 57.14% |  |  |
| Diabetes | Without | 12 | 8 | 40.00% | 10.989a | 0.001* |
|  | With | 2 | 18 | 90.00% |  |  |
| Nervous invasion | Without | 7 | 13 | 65.00% | .051a | 0.821 |
|  | With | 6 | 13 | 68.42% |  |  |
| Vascular tumor thrombus | Without | 4 | 6 | 60.00% | .147a | 0.702 |
|  | With | 10 | 20 | 66.67% |  |  |
| T | T1 | 0 | 2 | 100.00% | 4.224a | 0.238 |
|  | T2 | 1 | 4 | 80.00% |  |  |
|  | T3 | 11 | 12 | 52.17% |  |  |
|  | T4 | 2 | 8 | 80.00% |  |  |
| N | N0 | 2 | 7 | 77.78% | 2.793a | 0.425 |
|  | N1 | 3 | 8 | 72.73% |  |  |
|  | N2 | 6 | 5 | 45.45% |  |  |
|  | N3 | 3 | 6 | 66.67% |  |  |
| M | M0 | 14 | 25 | 64.10% | .552a | 0.457 |
|  | M1 | 0 | 1 | 100.00% |  |  |
| Differentiation | High | 9 | 9 | 50.00% | 3.237a | 0.072 |
|  | Poorly | 5 | 17 | 77.27% |  |  |

**Table S2 Hyperglycemic related genes**

ID UCP3 HNRNPUL2-BSCL2 PLIN1 MLKL APOE CDKAL1 MT-TK CELA2A CAT GAD2 AKR1B1 PTPRN C12orf43 GATA6 AGER CCR5 AGPAT2 IGF1 CRP IAPP IL1RN STAT3 DNAJC3 ITPR3 SUMO4 TNF LEPR HLA-DRB1 PRMT7 HLA-DQA1 GCG ALB MIR657 EIF2AK3 SOD2 EPO ALMS1 LEP IER3IP1 PLAGL1 SPINK1 BSCL2 HLA-DQB1 MC4R IL2RA PTF1A RFX6 MT-TE DCAF17 HMGA1 APPL1 SLC19A2 MT-TL1 GPD2 PON1 ADIPOQ CAPN10 HFE FOXP3 CTLA4 MAPK8IP1 PTPN1 TBC1D4 VEGFA LIPC PPP1R3A PTPN22 MTNR1B CEL ACE LMNA IRS2 ZFP57 SLC2A4 ENPP1 AKT2 IGF2BP2 GLIS3 BLK RETN AQP2 INS-IGF2 SLC30A8 KLF11 SLC2A2 AVPR2 AVP IRS1 TCF7L2 IL6 PPARG PAX4 NEUROD1 WFS1 PDX1 HNF1B INSR HNF4A HNF1A ABCC8 GCK KCNJ11 INS IRS4 PIK3R1 PIK3R2 PIK3R3 PIK3CA PIK3CD PIK3CB MAPK1 MAPK3 MTOR PRKCZ SOCS1 SOCS2 SOCS3 SOCS4 IKBKB MAPK8 MAPK10 MAPK9 PRKCD PRKCE MAFA HK3 HK1 HK2 HKDC1 PKM PKLR CACNA1C CACNA1D CACNA1A CACNA1B CACNA1E CACNA1G A2M ABCA1 ABCA4 ABCB1 ABCC5 ABCG1 ABCG2 ABCG5 ABCG8 ABHD5 ABHD6 ABO ACACA ACACB ACAD10 ACAT1 ACE2 ACHE ACLY ACOT1 ACP1 ACP7 ACSL1 ACTN4 ACVR2B ADA ADA2 ADAD1 ADAM10 ADAM17 ADAM30 ADAM9 ADAMTS13 ADAMTS3 ADAMTS5 ADAMTS9 ADAMTS9-AS2 ADAMTSL3 ADAR ADCY3 ADD1 ADGRG1 ADH1B ADH1C ADIPOR1 ADIPOR2 ADM ADPGK ADRA2A ADRA2B ADRB2 ADRB3 AFF3 AFM AFP AGBL1 AGMO AGR2 AGR3 AGT AGTR1 AGXT2 AHI1 AHSG AIM2 AIMP1 AKNAD1 AKR1B10 AKR1D1 AKT1 ALAD ALCAM ALDH1A1 ALDH1L1 ALDH1L1-AS2 ALDH2 ALDH6A1 ALDH7A1 ALDOB ALOX12 ALOX15 ALOX5 ALOX5AP ALPI ALPK1 ALPL AMBP AMH AMPD1 AMY1A AMY2A ANG ANGPT1 ANGPT2 ANGPTL2 ANGPTL3 ANGPTL4 ANGPTL6 ANGPTL7 ANGPTL8 ANK1 ANKH ANKK1 ANKRD34C-AS1 ANXA1 ANXA2 ANXA5 AOC3 AP3S1 AP3S2 APAF1 APELA APEX1 API5 APLN APLNR APOA1 APOA2 APOA4 APOA5 APOB APOC1 APOC2 APOC3 APOD APOH APOL1 APOM APP AQP11 AQP3 AQP5 AQP7 AQP9 AQR AR ARAP1 ARAP1-AS1 ARAP1-AS2 ARF5 ARG1 ARG2 ARHGAP18 ARHGAP4 ARHGEF11 ARHGEF12 ARHGEF7 ARID5B ARL15 ARL6 ARNT ARNTL2 ARPC3 ASCL2 ASIP ASL ASXL2 ATF2 ATF3 ATF4 ATF6 ATG16L1 ATM ATP1A2 ATP2A2 ATP2A3 ATP4A ATP5F1B ATP5PO ATP6AP2 ATP6V0A2 ATP6V1H MT-ATP8 ATRN ATXN2 AVPR1A AVPR1B AXL AZGP1 BACE1 BACH2 BAD BARX2 BBS10 BCAR1 BCHE BCL11A BCL2 BCL2L11 BCR BDKRB2 BDNF BECN1 BGLAP BHMT BHMT2 BID BIN1 BIRC5 BLVRA BMP1 BMP6 BNC2 BNIP3 BRAP BRCA1 BRCA2 BRINP3 BSG BTC BTN2A1 BTN3A1 BTNL2 C11orf65 C1QTNF1 C1QTNF12 C1QTNF3 C1QTNF6 C1QTNF9 C2CD4A C2CD4B C3 C3orf85 C4A C4B C5 C5AR2 C5orf67 C6orf120 C7orf50 CA1 CADM2 CALCA CALCRL CALD1 CALM2 CAMK1D CAMK2D CAMK2G CAMP CAPN1 CAPN13 CAPN2 CAPSL CARD8 CARM1 CASC15 CASC17 CASC2 CASP8 CASP9 CASR CAV1 CAV2 CBLB CBR3 CCAR2 CCDC92 CCL2 CCL23 CCL24 CCL25 CCL3 CCL4 CCL5 CCNL1 CCNQ CCR2 CCR9 CD101 CD14 CD163 CD1D CD226 CD24 CD247 CD274 CD276 CD2AP CD34 CD36 CD38 CD4 CD40 CD40LG CD44 CD48 CD52 CD59 CD69 CD74 CD86 CDC123 CDCA7 CDH1 CDH13 CDH16 CDK11A CDK2 CDK4 CDKN1A CDKN1C CDKN2A CDKN2B CDKN2B-AS1 CEACAM1 CEACAM8 CEBPA CELA1 CELA3B CELF1 CENPA CENPO CENPW CETN3 CETP CFD CFH CFI CFP CFTR CHD1L CHFR CHGA CHI3L1 CHIT1 CHN2 CHRM3 CHRNB4 CHTOP CIDEC CIITA CISD1 CISD2 CLCNKA CLDN1 CLDN4 CLEC16A CLPS CLRN1 CLSTN2 CLTCL1 CLTRN CLU CMA1 CMIP CMKLR1 CNDP1 CNDP2 CNKSR3 COBL COL18A1 COL1A1 COL3A1 COL4A2 COL5A1 COL8A1 COLEC10 COLEC12 COMT COQ5 CORIN CORT COX18 MT-CO2 COX7B2 CP CPA6 CPB2 CPEB2-DT CPS1 CPSF2 CPT1A CPVL CR2 CREB1 CREB3L3 CREBRF CRHR2 CRTC2 CRY2 CRYAA CRYAB CSF3 CSH1 CSK CSNK2A1 CST3 CTBP1-DT CTCFL CTF1 CTH CTNNB1 CTSB CTSC CTSD CTSH CTSL CTSS CTSV CUBN CX3CL1 CXCL1 CXCL10 CXCL12 CXCL14 CXCL16 CXCL2 CXCL5 CXCL8 CXCL9 CXCR1 CXCR3 CXCR4 CYB5B CYBA CYCS CYP11B2 CYP17A1 CYP19A1 CYP1A1 CYP1A2 CYP24A1 CYP27B1 CYP2A6 CYP2C19 CYP2C9 CYP2D6 CYP2E1 CYP2J2 CYP2R1 CYP3A4 CYP3A5 CYP46A1 CYP4F2 CYP7A1 CYP7B1 DACH1 DACT1 DANCR DAPK1 DAPK3 DBI DBP DCD DCDC2C DCN DDAH1 DDAH2 DDC DDIT4 DDOST DDX21 DDX39B DDX58 DEAF1 DEFA1 DEFA3 DEFB1 DEFB127 DEXI DGAT1 DGAT2 DGKB DGUOK DHCR7 DHX35 DICER1 DIO2 DIO3 DKK1 DLG4 DLK1 DLL1 DMC1 DMGDH DMPK DNAH10 DNAH2 DNAJC27 DNASE1 DNER DNMT1 DNMT3A DOK1 DOK5 DPP4 DPP9 DPYSL3 DRD2 DRD3 DROSHA DUSP1 DUSP10 DUSP12 DUSP9 DUT E2F1 ECE1 EDA EDEM2 EDN1 EDNRA EDNRB EEA1 EFR3B EGF EGFR EGR1 EIF2AK2 EIF2B1 EIF4A2 EIF4E ELANE ELAVL1 ELMO1 ELN ELOVL5 EMX2 ENG ENGASE ENHO ENO2 ENPP2 ENSA ENTPD1 EP300 EPHA7 EPHX1 ERBB2 ERBB3 ERBB4 ERG ERO1A ESAM ESM1 ESR1 ESR2 ESRRA ESRRG ETS1 ETV1 ETV5 EXOC4 EXOG EXT2 EZH2 EZR F12 F13A1 F13B F2 F2RL3 F3 F5 F7 F8 F9 FAAH FABP1 FABP2 FABP3 FABP4 FABP5 FABP6 FADD FADS1 FADS2 FADS3 FAF1 FAIM2 FAM120B FAM241B FANCC FAP FASLG FASN FAT3 FBLN1 FBN1 FBP1 FBXW7 FCGR2A FCGR3A FCN1 FCN3 FCRL3 FDFT1 FDX1 FECHP1 FEN1 FETUB FFAR2 FFAR3 FGA FGB FGF1 FGF19 FGF2 FGF21 FGF23 FGG FGL1 FITM2 FKBPL FLT1 FLT4 FLVCR1 FN1 FN3K FNDC5 FOLH1 FOSL2 FOXC1 FOXC2 FOXM1 FOXO1 FOXO3 FOXP2 FPR2 FRMD3 FRZB FST FSTL3 FTH1 FTL FTO FUCA1 FURIN FUT2 FUT6 FXN FYB1 FZD4 G6PC2 G6PC3 G6PD GAA GAB3 GABPA GABRA4 GADD45A GAL GAL3ST1 GALNT14 GALNT2 GANC GAPDH GAR1 GAS2 GAS5 GAS6 GAST GATA3 GATA4 GC GCA GCC1 GCH1 GCKR GCLC GCLM GCNT1 GDF15 GDF2 GFER GFPT1 GFPT2 GGA3 GGT1 GH1 GHR GHRL GHSR GIGYF1 GIP GJA4 GJC1 GLB1 GLCCI1 GLI2 GLO1 GLP1R GLP2R GLRA3 GLS GNAS GNAT3 GNB1 GNB3 GNL3 GORAB GOT1 GOT2 GP1BA GP2 GPAT4 GPATCH2 GPBAR1 GPD1 GPER1 GPIHBP1 GPLD1 GPNMB GPR21 GPRC5B GPS2 GPSM1 GPT GPX1 GPX2 GPX3 GPX4 GRB10 GRB14 GRK2 GRK4 GRK5 GRN GSC GSDMB GSDMC GSK3B GSTA1 GSTK1 GSTM1 GSTP1 GUCA2A GUCA2B GYS1 GZMB H19 HACD3 HADH HAMP HAS2 HAVCR1 HAVCR2 HBA1 HDAC3 HDAC4 HDAC5 HDAC9 HECTD4 HECW1 HERPUD2 HES1 HEXB HGF HHEX HIF1A HIF3A HINT1 HLA-A HLA-B HLA-C HLA-DMA HLA-DMB HLA-DOA HLA-DPA1 HLA-DPB1 HLA-DQA2 HLA-DRA HLA-DRB5 HLA-E HLA-G HM13 HMG20A HMGA2 HMGB1 HMGB1P1 HMGCR HMOX1 HNRNPD HP HPSE HPSE2 HRAS HRH4 HS6ST3 HSD11B1 HSD11B2 HSD17B12 HSD17B14 HSD3B1 HSF1 HSFX1 HSP90AA1 HSP90B1 HSPA1A HSPA1L HSPA4 HSPA5 HSPA8 HSPB1 HSPB2 HSPD1 HSPE1 HTR1A HTR2A HTR2C HUNK HYAL1 ICA1 ICAM1 IDE IDO1 IFIH1 IFNA1 IFNB1 IFNL3 IGF1R IGF2 IGF2-AS IGF2BP1 IGF2BP3 IGF2R IGFBP1 IGFBP2 IGFBP3 IGFBP4 IGFBP5 IGFBP7 IGHE IKZF1 IKZF4 IL10 IL12B IL12RB2 IL13 IL15 IL16 IL17A IL17RA IL18 IL19 IL1A IL1B IL1RAP IL1RL1 IL1RL2 IL2 IL20 IL20RA IL21 IL21R IL22 IL23A IL23R IL25 IL27 IL32 IL33 IL36A IL36G IL4R IL6R IL6ST IL7 IL7R IL9 ILF3 IMPDH2 INAFM2 INHBA INPPL1 INSIG1 INSIG2 INSM1 INSM2 INTS8 IP6K1 IPPK IRAK1 IRAK4 IRF5 IRF8 IRX3 IRX5 ISCA1P1 ITCH ITGA1 ITGA2 ITGA2B ITGAL ITGAM ITGB1 ITGB2 ITGB3 ITGB6 ITLN1 JAK1 JAZF1 JAZF1-AS1 JMJD1C KAT2B KCNA3 KCNIP1 KCNJ15 KCNJ5 KCNJ8 KCNK16 KCNK17 KCNMA1 KCNQ1 KCNQ1OT1 KDM4C KDM6A KDR KEAP1 KIAA1109 KIF11 KIF17 KIF6 KIFC1 KIR2DL1 KIR2DL3 KIR2DS4 KIR3DL1 KIRREL1 KIRREL2 KL KLF14 KLF2 KLF4 KLF5 KLF7 KLF9 KLHL1 KLHL2 KLHL22 KLHL29 KLK1 KLK3 KLRC3 KLRD1 KLRK1 KRAS KRT18 KSR2 LAG3 LAMA1 LARP6 LARS2 LATS2 LBP LCAT LCK LCN2 LCT LDHA LGALS1 LGALS13 LGALS2 LGALS3 LGALS3BP LGALS9 LGMN LGR5 LIAS LILRB1 LIN28A LINC01104 LINC01214 LINC01339 LINC01584 LINC01734 LINGO2 LIPE LIPG LIPH LMO7 LMX1A LNPEP LOX LPA LPAL2 LPAR3 LPIN1 LPIN2 LPL LPP LRBA LRG1 LRIG1 LRIG2 LRIG3 LRP1 LRP2 LRP2BP LRP5 LRP6 LRRC7 LTA LTF LY75 LY9 LYPLAL1 MACF1 MADD MAEA MAGI1 MALAT1 MALT1 MAN2A1 MANF MAOA MAOB MAP3K1 MAP3K14 MAP3K5 MAP4K4 MAP4K5 MAPK14 MAPK7 MAPT MASP1 MASP2 MAVS MBL2 MBL3P MBOAT4 MC3R MC5R MCAM MCF2L2 MCL1 MCM6 MCTP2 MDK MDM2 MEDAG MEF2C MEFV MEG3 MEG8 MEN1 MEOX2 MERTK MEST METRNL MFGE8 MFN2 MFSD2A MGLL MGMT MGP MIA3 MICA MICB MIF MIR100 MIR101-1 MIR101-2 MIR10B MIR126 MIR1285-1 MIR1285-2 MIR1303 MIR130A MIR132 MIR133B MIR134 MIR140 MIR146B MIR149 MIR150 MIR152 MIR154 MIR15B MIR16-1 MIR16-2 MIR181A1 MIR181B1 MIR181B2 MIR182 MIR185 MIR190A MIR194-1 MIR196A2 MIR199A1 MIR200B MIR200C MIR203A MIR204 MIR210 MIR216A MIR217 MIR218-1 MIR218-2 MIR222 MIR23A MIR23B MIR2467 MIR27A MIR27B MIR30D MIR31 MIR3135B MIR3188 MIR320A MIR33A MIR340 MIR342 MIR34C MIR3681HG MIR370 MIR375 MIR377 MIR410 MIR425 MIR429 MIR450A1 MIR455 MIR4739 MIR483 MIR486-1 MIR499A MIR520H MIR550A1 MIR550A2 MIR571 MIR574 MIR593 MIR646HG MIR661 MIR770 MIR802 MIR892B MIR9-1 MIR93 MIR933 MIR96 MIRLET7A1 MIRLET7G MLST8 MME MMP1 MMP10 MMP12 MMP13 MMP14 MMP2 MMP3 MMP7 MMP8 MMP9 MOGAT2 MORC1 MPHOSPH9 MPL MPO MPP2 MRPS31 MS4A1 MSH2 MSN MT1A MT1F MT2A MTHFD1 MTHFR MTNR1A MTRR MTSS1 MTTP MUC1 MUC5B MUSK MUTYH MYH9 MYL2 MYLK MYO9B NAA25 NAMPT NANOG NAT2 MT-ND1 MT-ND2 MT-ND4L MT-ND5 NDRG1 NDUFB6 NEAT1 NEGR1 NEIL1 NEK7 NF1 NFAT5 NFATC2 NFATC3 NFATC4 NFE2L2 NFE2L3 NFKB1 NFKBIA NFKBIL1 NFYA NKX2-5 NKX6-1 NLRP1 NLRX1 NMU NNAT NNMT NOD1 NOD2 NOS1AP NOS3 NOTCH1 NOTCH2 NOTCH3 NOX1 NOX4 NOX5 NOXO1 NPC1L1 NPHS1 NPHS2 NPPA NPPB NPPC NPR2 NPY1R NPY2R NQO1 NR1D1 NR1D2 NR1I2 NR3C1 NR4A2 NR4A3 NR5A1 NRF1 NRG1 NRG3 NRG4 NRP1 NSA2 NT5C2 NTRK2 NTS NUAK2 NUCB1 NUCB2 NUDT1 NUS1 NXN OAS1 OASL OCLN OFCC1 OGA OGG1 OGN OIP5-AS1 OLR1 ONECUT1 OR12D3 ORAI1 ORMDL3 OSM OSTN OXT OXTR P2RX1 P2RX7 P2RY12 P2RY2 PAIP1 PAK1 PAM PAOX PAPPA PARK7 PARL PARP1 PARP11 PARP2 PASK PAX3 PBRM1 PBX1 PC PCBD1 PCBD2 PCBP2 PCCA PCDH18 PCK2 PCLO PCNT PCNX2 PCSK1 PCSK9 PDCD1 PDCD5 PDE10A PDE3A PDE3B PDE5A PDGFA PDGFB PDK1 PDK2 PDK4 PDLIM5 PDZRN3 PEA15 PECAM1 PEMT PENK PEPD PER3 PEX5L PF4 PF4V1 PFKFB3 PGC PGF PGM1 PGR PHACTR2 PHEX PHF10 PHGDH PHLPP1 PHLPP2 PHOSPHO1 PHTF1 PI16 PICALM PIK3C2A PIK3C2G PIK3CG PIN1 PINK1 PITPNC1 PKN2 PLA2G2A PLA2G4A PLA2G6 PLA2G7 PLAT PLAUR PLEKHA1 PLG PLIN2 PLIN3 PLK1 PLS1 PLTP PLXNA4 PLXND1 PNPLA3 PODXL POLG POLI POLR2G POMC POMGNT1 PON2 PON3 POR POSTN POU2F1 POU5F1 POU5F1B PPARD PPARGC1A PPARGC1B PPBP PPDPF PPIA PPIE PPM1F PPM1K PPP1CA PPP1R12A PPP1R13L PPP1R15B PPP1R3B PPP2R2A PPP2R2C PPRC1 PPY PRC1 PRC1-AS1 PRDM16 PRDM5 PRDX4 PRDX6 PREX1 PRKAB2 PRKAG2 PRKCA PRKCB PRKCSH PRKD1 PRKD2 PRL PRLR PRM3 PRNP PROC PROM1 PROS1 PROX1 PROX1-AS1 PRRC2A PRSS1 PRTN3 PSD3 PSMA6 PSMB1 PSMB8 PSMD6 PSMD9 PTEN PTGES2 PTGIR PTGIS PTGS1 PTGS2 PTH PTH1R PTHLH PTMA PTPN2 PTPRC PTPRD PTPRF PTPRM PTPRN2 PTPRS PTX3 PVT1 PYGL PYY QRFP R3HDML RAB11FIP2 RAB38 RAB5B RAB5C RAB8A RAC1 RAD51 RAD51B RAD51C RAF1 RALGAPA2 RAN RAPGEF1 RAPGEF5 RARRES2 RASGRP1 RB1 RBFOX1 RBM38 RBM43 RBMS1 RBMS2 RBP4 RBPJL RCN2 RD3 RDX REG1A REG1B REG3A RELA REN RGMA RGN RGS2 RGS7 RHD RHOA RHOU RIN3 RLN2 RLN3 RNASEH1 RNASEK RND3 RNF10 RNF6 RNLS MT-RNR2 ROCK2 ROMO1 RORA RPH3A RPS6KA6 RPS6KB1 RPSAP52 RPSAP9 RRAD RREB1 RTL1 RUNX3 RXRA RXRG RYR3 S100A12 S100A13 S100A4 S100A8 S100A9 S100B SAA1 SACS SATB2 SCAF8 SCARB1 SCD SCG5 SCP2 SCUBE1 SCUBE2 SDC1 SDC2 SDF2 SDF2L1 SDHAF4 SELE SELENOP SELENOS SELP SERP2 SERPINA1 SERPINA12 SERPINA4 SERPINA7 SERPINB2 SERPINC1 SERPINE1 SERPINF1 SERPINF2 SESN2 SETD7 SFRP4 SFRP5 SFTPD SGCG SGK1 SGSM2 SH2B3 SH2D4A SH3BP4 SH3YL1 SHBG SHC1 SI SIAE SIGLEC5 SIGLEC7 SIRPG SIRT1 SIRT2 SIRT6 SKAP2 SKP2 SLAMF1 SLC10A2 SLC11A2 SLC12A3 SLC16A1 SLC16A11 SLC16A13 SLC16A4 SLC17A9 SLC19A3 SLC22A1 SLC22A11 SLC22A2 SLC22A23 SLC22A3 SLC22A4 SLC22A5 SLC23A2 SLC24A4 SLC25A36 SLC25A4 SLC26A6 SLC26A9 SLC29A1 SLC29A3 SLC2A1 SLC2A10 SLC2A12 SLC2A3 SLC2A9 SLC39A10 SLC39A6 SLC3A2 SLC40A1 SLC44A3 SLC47A1 SLC4A4 SLC5A1 SLC5A2 SLC5A3 SLC6A12 SLC6A13 SLC6A2 SLC6A20 SLC6A4 SLC6A6 SLC7A1 SLC9C1 SLCO1B1 SLMAP SMAD2 SMAD3 SMAD7 SMIM2-AS1 SMOC1 SNAI2 SNAP23 SND1 SNHG17 SNX19 SNX29 SNX7 SOAT1 SOD1 SOD3 SORBS1 SORCS1 SORD SORL1 SORT1 SOS1 SOST SOX13 SOX2 SOX4 SOX6 SP1 SP3 SPAG16 SPARC SPHK1 SPON2 SPP1 SPRY2 SPTLC3 SPX SRC SREBF1 SREBF2 SRR SRRM1 SRSF1 SSR1 SST ST6GAL1 STAC STAT1 STAT4 STAT5A STC2 STEAP1B STEAP4 STK11 STK4 STRA6 STX1A STX4 STX8 STXBP2 SUCNR1 SULT4A1 SUOX SUV39H1 SUV39H2 SVEP1 SVIP SYK SYN2 TAB2 TAC1 TADA2A TAF1C TAF5L TAGAP TAGLN2 TALDO1 TAP1 TAP2 TAS2R9 TBL1X TBP TBXA2R TCERG1L TCF19 TEK TENM4 TERT TET2 TF TFAP2B TFB1M TFPI TFPI2 TFRC TGFB1 TGFB3 TGFBI TGFBR1 TGFBR3 TGFBRAP1 TGM2 THADA THBD THBS1 THBS2 THBS4 THRA THRAP3 TIAM1 TIGIT TIMD4 TIMM44 TIMP1 TIMP3 TIRAP TK1 TKT TLE1 TLE3 TLE4 TLL1 TLN1 TLR1 TLR10 TLR3 TLR5 TLR6 TLR9 TMEFF2 TMEM154 TMEM175 TMEM18 TMEM45B TMPRSS2 TMPRSS6 TNFAIP3 TNFRSF11A TNFRSF11B TNFRSF1A TNFRSF1B TNFSF10 TNFSF11 TNFSF12 TNFSF13 TNFSF13B TNFSF14 TNFSF4 TNMD TNNI3 TNNT2 TNP2 TNRC6A TOMM40 TOR2A TOX TP53 TP53INP1 TP53INP2 TPCN2 TPI1 TPM1 TPM4 TPO TPSAB1 TRAF6 TRBV20-1 TREM1 TREM2 TRH TRIB1 TRIB2 TRIB3 TRMT10A MT-TT TRPC1 TRPC6 TRPM2 TRPM5 TRPM6 TRPM7 TRPV4 TSC2 TSC22D2 TSC22D3 TSC22D4 TSHZ1 TSPAN12 TSPAN6 TSPAN7 TSPAN8 TTC28-AS1 TTR TWIST1 TWNK TXN TXN2 TXNDC5 TXNIP TXNRD1 TXNRD2 TYK2 TYRO3 UBASH3A UBE2E2 UBE2O UBE2Z UCHL1 UCN3 UCP2 UGT1A1 UGT1A10 UGT1A3 UGT1A4 UGT1A5 UGT1A6 UGT1A7 UGT1A8 UGT1A9 UGT2B15 UGT2B7 UMOD UNC13B USF1 USP21 USP36 UTS2 UTS2B VASP VCAM1 VDR VEGFC VIM VIM-AS1 VIPR1 VNN1 VPS13C VPS26A VPS33B VPS39 VRK1 VTCN1 VTN VWA2 VWF WDR19 WDR27 WDR72 WIF1 WNK4 WNT5A WNT5B WNT9B WRN WWOX XDH XRCC1 XRCC3 XYLT1 YAP1 YES1 YIPF5 YTHDF2 ZBED3 ZBED3-AS1 ZEB1 ZFAND3 ZFAND6 ZFP36 ZFP36L1 ZMAT4 ZMIZ1 ZNF410 ZNF608 ZNF648 ZNRF1 ZPLD1 ZPR1 ZRANB3 ZYG11A

**Table S3 Primers used in this study.**

| ACLY FORWARD | 5'CAGAATCGGTTCAAGTATGCTC3' |
| --- | --- |
| ACLY REVERSE | 5'AAGTTTTCCACGACGTTTGATC3' |
| si-NC sense | 5'UUC UCC GAA CGU GUC ACG UTT3' |
| si-NC antisense | 5'ACG UGA CAC GUU CGG AGA ATT3' |
| si-ACLY sense | 5'CGU GAG AGC AAU UCG AGA UUA3' |
| si-ACLY antisense | 5'UAA UCU CGA AUU GCU CUC ACG3' |
| Gapdh sense | 5′- TGGCAAAGTGGAGATTGTTGCC -3′ |
| Gapdh antisense | 5′- AAGATGGTGATGGGCTTCCCG -3′ |
